# Extra Supplement—The Nursing Mirror

**Published:** 1889-02-09

**Authors:** 


*The Hospital,*
FEBRUARY 9, 1889.

[*Extra Supplement.*

### The Nursing Mirror.

All Contributions for this Supplement should be addressed "The Nursing Mirror," care of The Editor, 38, Old Jewry, E.C.

### En Passant.

NEWS FROM THE PROVINCES.—The Frome Home for Trained Nurses issues a satisfactory annual report. One hundred and fifty-two cases were attended last year, the average number of visits to one case being 29. The only blot in the cheerful report is that the attempt to provide a maternity nurse proved a failure. Miss Briggs is still superintendent.—The Leeds District Nursing Society also progresses well. The seven nurses attended 744 patients last year, but here also the Committee have decided to relinquish the maternity branch.—At the annual meeting of the Shaftesbury Hospital, Dorset, the Rev. F. Ehlvers proposed that district nurses should be provided for that neighbourhood, and Dr. Evans warmly seconded the motion, which he said he considered a most important one. The matron of Shaftesbury Hospital, Miss Wand, also had a special vote of thanks moved in her favour by Canon Glyn.—On January 31st Thomas Vicars, Esq., F.G.S., gave an instructive and interesting lecture on "Electricity" to the nurses of Torbay Hospital. Here is an example to be followed.

WOMEN'S WAGES.—Mrs. Fawcett lately delivered an excellent lecture on the subject, "Why are Women's Wages Lower than Men's?" Mrs. Fawcett was very fine on the nursing side of the question, and spoke of the example of thoroughness shown by Miss Nightingale. If a woman works thoroughly in a trained, useful manner, she ought to be thoroughly well paid. Mrs. Fawcett gave three reasons for the prevailing lowness of women's wages: (1) The fewer trades open to them as compared to those open to men. (2) Lack of thorough training in women. This is often due to parents objecting to spend so much on a girl's as on a boy's education or apprenticeship. Also that women object to spending a long time in learning a trade, and therefore there are few who take up wood engraving, which needs five years to learn properly. (3) The greater use of machinery, which decreases fine handwork such as women excel in. Mrs. Fawcett also spoke very strongly on the necessity for women workers to pay attention to their food; they could not compete with men if they insisted on living chiefly on tea and buns. We think Mrs. Fawcett would approve of "coaxing the appetite."

UNINTELLIGENT NURSING.—Those doctors who are yet foolish enough to give their preference to uneducated nurses ought to understand to what extra care and intelligence they bind themselves. The fatal misadventure which occurred at University College Hospital last week would never have happened had the nurse read and understood the written prescription. The house surgeon put on the order board, "22 grs. of cocaine in an ounce of water; statim." To every nurse the abbreviation "stat." or the full word "statim" means simply "immediately." This is the explanation of it given in "Domville," and every standard work on nursing. The nurse took the board down to the dispensary at once, and received a liquid in a measuring glass. When she returned to the ward the surgeon was not there, and she naturally administered the solution as a draught. The patient died. The surgeon, of course, meant to use the cocaine as a hypodermic injection, but he did not write "injection" on the board, and in the meantime he was called away. He contended he would have written "sumendum" if he wished the medicine administered. We cannot see that the nurse was to blame in this case, but still many nurses would have known that to administer 22 grains of cocaine meant death to the patient. So long as nurses are not all taught the elements of pharmacy and therapeutics, the physician must be particularly careful to give full and plain directions about all drugs ordered. We give this week a list of abbreviations every nurse should know.

THE FIRST THOUSAND.—We should like to write an ode in honour of the first thousand nurses who join the National Pension Fund, only unluckily we are not Tennyson, nor even one of the minor poets. In default of verse we must therefore state in prose that we think those nurses who in face of systematic opposition have had the courage and clear-headedness to join an Association which offered them business advantages—not charity—are to be highly commended. Surely the council of the Pension Fund will recognise this, and just as surely will they give honour where honour is due. Several institutions are now considering whether they shall follow the example of Mildmay, and join *en masse*, so that any day the limit of the first thousand may be reached, and it may be too late to be numbered amongst the lucky pioneers. In a few years when time has swept away the evil feelings which now animate a tiny section of the nursing world, it will be the glory of a nurse to state: "I was one of the first thousand!"

WORKHOUSE NURSING.—The ninth report of the Workhouse Infirmary Nursing Association is just out, and tells of steady growth. The situations to which probationers were appointed during 1888 were the following: St. George's-in-the-East Infirmary, seven night nurses; Whitechapel Infirmary, one assistant and one night nurse; Paddington Infirmary, one night nurse; St. Pancras Workhouse (new wing), one day nurse; Isle of Thanet Union, at Minster, one head nurse; Stamford Union, one nurse; Elham Union, Folkestone, nurse and midwife; Beckham Union, nurse and midwife; Portsmouth Union, one midwife. The names of Lady Knutsford and Mrs. Bonham Carter have been added to the committee. Finances are satisfactory, but still the Boards of Guardians who benefit by this association should subscribe in greater numbers. The total number of nurses belonging to the association is now 91.

THE DUKE OF WESTMINSTER.—On January 29th, at the annual meeting of the Chester Infirmary, the Duke of Westminster, who was presiding, in moving the adoption of the report and statement of accounts, referred to the fact mentioned in the report that the Misses Wilbraham were unable to continue their support to the district nurses in two of the parishes of Chester. He would ask whether some more able and systematic arrangement could be come to with regard to the district nursing through the whole town? His attention had for many years been called to this subject in connexion with the National and Metropolitan Nursing Institution in London—for nursing the poor in their own homes. This principle had had a great stimulus by the donation on the part of her Majesty of the surplus of the Jubilee fund, for the establishment of district nursing in the United Kingdom under Royal Charter, which would shortly be granted. They had a committee for England and Scotland, and were in the throes of a committee for Ireland, owing to the difficulty of religion and nationalism, which was very great where the various religions came into contact. He believed that the deaconesses in Chester were doing a great work, but he did not know whether the work was being done in so general and systematic a manner as many of them would like to see. He thought some attention should be given to this point, because the medical men who were present knew how greatly they were assisted by the nursing of women and ladies well qualified to nurse the poor in their own homes, thus prolonging life and in other ways being of infinite benefit. There was another matter, that of the increase in the number of fever patients. He understood that by building accommodation for the nurses outside the fever hospital sufficient accommodation for all ordinary requirements, and even for an increase in the number of fever patients, could be made. He had heard the matter mooted, and if it could be done it would be a great thing.

### Original Articles.

### CASE NUMBER NINE.

"Above all you will see that she is kept perfectly quiet," and the fussy little doctor, having concluded his directions, was off like a flash of lightning, leaving the nurse, a tall, stately woman, alone with the patient.

The "case" had been brought into the accident ward some hours previously, and had not shown consciousness until, within the last few moments, when she had suddenly opened her dark eyes on the doctor's anxious face. There was a silence after the nurse seated herself, her eye noting the short, quick breathing, which told that the patient was awake.

"Is there anyone near?" It was a feeble voice which broke the stillness, but showed unmistakable culture; "these bandages about my head are very tight; could they be made looser?"

Skilful fingers were deftly loosening the bandages, when a cry broke from the nurse's lips.

"What is it? oh! what?" weakly asked the patient.

"Nothing—that is, I feared I had hurt you." The stately nurse spoke calmly; she had regained her self-possession, thanks to her training. The word of command: "You will see that she is kept perfectly quiet," was ringing in her ear.

"Now, that is done, I think you will feel more comfortable."

As nurse turned to resume her seat, she was trembling in every limb, and her fair, placid face had gone white to the lips. So agitated was she, that the swift, searching look from the dark eyes on the pillow was unnoticed. There was another silence; the patient lay very still, but the nurse's fingers twitched convulsively.

"Haven't you found me out, yet, Vi?"

It was only a weak whisper, but it thundered through the nurse's brain. Twice her white lips parted to speak:

"Yes, dearest, I know it's you," at length the words came, but half-choked in the struggle to be calm. "See, dear; drink this and lie quite still for an hour, while I attend to the dinners. Then I will come and talk it all over." So nurse got away and fought down her emotion alone, and presently, in the quiet of the afternoon, returned to "case number nine" once more.

"Oh, Essie! At last—at last I have found you—my poor little sister."

"Hush, Vi, you must be calm, for my sake."

"Speak then, Essie," and Vi sat upright, forcing down her tears; "tell me all, if you can. But, first, about this accident. You were thrown out —— '

"Yes, yes. The omnibus must have fallen over. I don't remember anything more, it got so dark. Was I hurt? Did they bring me to this place?"

"Yes, dear. It was a fearful thing. An omnibus, overcrowded, lurched over, and many were injured. There was great excitement over such an unusual accident. They brought you in last night, but I did not see you until this morning, and then I didn't recognise you; it was only when I altered the bandages that I knew it was our long-lost Essie. Dear one, how we have sought for you all this long time. Why did you hide away from us?"

"Why? Because of father's anger. He cast me off—you know it—when I married Frank."

"But he repented his harshness; he wanted you back. Did you never see the advertisement he put in the news papers?"

"Never. Oh, Vi, my life has been a sore one ever since I left you."

"Where is *he*?" The question came from sternly-tightened lips.

"Frank? Gone away, I don't know where. He deserted me. It has been a weary time of sorrow. I was never happy; I never deserved to be after defying my father. It was a disgrace for his daughter to run away with an actor; as a clergyman he felt it more than another man would have done. People haven't such a horror of the stage nowadays, but father is one of the old school and abhors it. No, I am not exerting myself too much, dear old Violet; it does me more good than anything else could to have you close by me listening to it all. After the first year we were wretchedly unhappy; he grew tired of me, and made me feel I was a drag upon him. And he hated our little child —— "

"Is there a child? Oh, Essie, and I never knew!" Vi's face flushed in her eager surprise.

"There *was*." Essie's voice sank to a whisper. "Oh, Vi, dear old sister, that is the bitterest, cruellest wrong of all; think what it was to have a little creature of your very own, to feel her tiny fingers straying over your face, and to see her blue eyes watching your movements about the room, and—then to lose her."

"It died then?"

"She was killed—killed—he did it."

"Oh, Essie, hush, you are delirious, you are wandering. Oh, what will Dr. Simcox say?"

"No, Vi," and the patient spoke more calmly, "it is no question of delirium. Listen! I can speak of it now, quietly. It happened one evening; he was ready to go to the theatre to his work. I asked him for money—we were hungry, the child and I—he kept us very short of even necessary food. He flew into a passion, and, for the first time, he struck me. The child was in my arms, and I fell to the floor with her, the little head striking on the corner of the fender. I suppose I fainted. When I became conscious I was lying as I fell, and she—my blue-eyed baby—there was a cruel mark on the veined temple. She was dead! We were alone in the room. He had left us there, and I have never seen his face since. What followed seems a dull dream. People came, hearing my shrieks, and brought a doctor. Then there was a long blank; I was very ill." Essie ceased speaking wearily, and Vi, with one hand catching wildly at her throat to keep down the agonised sobs, with the other clasped the little thin hand tighter. "Since that," began Essie again, "I have worked for bread—you remember how I used to sew all that dainty art-work, and how proud you were because I did it so well. My only accomplishment did me good stead; the shops gave me very little money, still, it brought me bread. When this accident happened to the omnibus, I was coming back from one of the West End shops which takes my work, and—it was a horrible accident——"

"But it has brought us together, Essie; you remember the old hymn—

'God moves in a mysterious way,
His wonders to perform.'

And now we have found you, father and I will never let you go, little sister."

"Tell me about father," Essie said feebly.

"He is sadly broken down—so much so, that I had made up my mind to throw up my profession, dear as it is to me, in order to go down to the vicarage and take care of him. You know since our mother's death he was never like other men; and then, your—your leaving home—— "

"Oh yes, yes, I know; but I've paid sorrowfully for my disobedience." The dark, sad eyes were beginning to blaze, and a faint pink for the first time stole into the white cheeks, at sight of which Violet's professional instincts be came on the alert.

"Now, little one," she spoke cheerily, "we're nurse and patient, if you please; I've got to make you strong and well in a very short time, and then we'll send you down to take care of father? And now, what you've got to do is to sleep, sleep, sleep, and forget."

It was marvellous, said everyone, to note the rapid progress made by Number Nine. Each day she made a clear stride towards recovery. When the bandages were removed the little head with the short dark curls looked surprisingly pretty; and Vi, the assiduous nurse, hung over the new-found sister with adoring eyes.

"She'll do now, my dear," whispered little Dr. Simcox confidentially, one day. The good old man had known the sisters from their childhood, and had been let into Essie's secret. "Get your father up as soon as possible," he added, "to take her down to the country to her old home."

In the old vicarage Essie moved about, at first with slow, halting steps. Reinstated in her old place in the thankful father's heart, she crept back, tended lovingly, to health and peace of mind. The sweet country air, the scented pine-woods round the old home, the village interests, all were the best of medicines for the hapless girl mentally and physically. The lurid past grew dim and pale; time had begun its healing work.

On her frequent breathless little visits down from the hospital, Vi thanked God unceasingly, that in His inscrutable wisdom He had brought the strayed lamb home to the fold, even though it had been by the *via dolorosa*—the way of sorrow.

### Common Abbreviations.

The following is a list of those abbreviations and Latin words, used in prescribing, which every nurse should understand:—

Ad effect. (Ad effectum)—Until effectual.
Ad lib. (Ad libitum)—At pleasure.
Alt. noct. (Alternâ nocte)—Every other night.
Alt. horis Alternis horis)—Every other hour.
Alt. dieb. (Alternis diebus)—Every other day.
A. c. (Ante cibum)—Before food.
Baln. tep. (Balneum tepidum)—Tepid bath.
B. d. (Bis die)—Twice a day.
Cat. (Cataplasma)—A poultice.
C. m. (Cras mane)—Every morning.
C. n. (Cras nocte)—Every night.
Coch. parvum (Cochleare parvum)—A tea-spoon.
Coch. magn. or amp. (Cochleare magnum)—A table-spoon.
Collyr. (Collyrium)—An eye wash.
Dext. lat. (Dextrum latus)—Right side.
Dexter (Dexter) - Right hand.
Dosis (Dosis)—A dose.
Emp. lyttæ (Emplastrum lyttæ)—A blister.
Gossypium (Gossypium)—Cotton wool.
Gutta (Gutta)—A drop.
H. n. (Hâc nocte.)—To-night.
Hor. decub. (Hora decubitus)—At going to bed.
Hor. som. (Horæ somni)—At going to sleep.
2dis horis (Secundis horis)—Every two hours.
6tis horis (Sextis horis)—Every six hours.
M. pr. (Mane primo)—First thing in the morning.
Misce (Misce)—Mix.
M. p. a. (Modo prescripto applicandum)—To be applied in the prescribed manner.
N. mque. (Nocte maneque)—Night and morning.
O. m. (Omni mane)—Every morning.
O. n. (Omni nocte)—Every night.
O. alt. hor. (Omnibus alternis horis)—Every other hour.
O. bid. (Omni biduo)—Every two days.
O. bih. (Omni bihora)—Every two hours.
P. a. a. (Parti affecti applicandum)—Apply to the affected part.
P. d. a. (Parti dolenti applicandum)—Apply to the painful part.
Part æq. (Partes æquales)—Equal parts.
P. c. (Post cibum)—After food.
Post sing. liq. sedes (Post singulas liquidas sedes)—After every loose motion.
P. r. n. (Pro re natâ)—As required.
P. m. (Primo mane)—First thing in the morning.
Pone Aurem (Pone Aurem)—Behind the ear.
S. (Sumendum)—To be taken.
Sinister (Sinister)—Left hand.
S. o. s. (Si opus sit)—If necessary.
Stat. (Statim)—Immediately.
T. d. s. (Ter die sumendum)—To be taken three times daily.
T. u. (Tussi urgente)—When the cough is troublesome.
Troch. (Trochiscus)—A lozenge.
U. (Utendum)—To be used.
Vesp. (Vespere)—In the evening.
Vic. (Vices)—Turns.

### Nurses as Stewardesses.

### THE PROS AND CONS.

We have received a great number of replies to our suggestion of opening a register for nurses who desire to become stewardesses, and as it is impossible to answer each letter separately, we propose to tell here what is desirable in such candidates. First we must remind our readers that the duties of a stewardess are as full of thorns as every other walk in life, and that those who would take that position must be women of courage, whose patience and gentleness is always unfailing. A stewardess has to attend to all lady passengers who are unwell, and make herself as useful and agreeable to them as she can. She is paid about £2 10s. a-month, and her berth, which she shares with other stewardesses, is usually over the screw. Now if, in spite of this unflattering picture, any of our correspondents hold to their original intention, will they please send us the following particulars, concisely written in their own handwriting, and addressed to "The Nursing Mirror:" (1) Name in full. (2) Age. (3) Where trained, and what certificate held. (4) Previous occupation before becoming a nurse. (5) Number of years engaged in nursing. (6) If a good sailor. (7) Single or widow. (8) Permanent address.

If the large shipping companies see the advantages which may accrue to them by this plan, it is hoped that they will carry a trained nurse on every large ship, just as certainly as they carry a doctor. At present the occupation of stewardess seems to descend from mother to daughter, and many of those who have acted in this capacity for years become known to constant travellers. To delicate ladies the presence of a trained nurse in her neat uniform, and with her skilful ways, would be a great blessing. Only nurses must not undertake this life "just for the fun of the thing;" the companies will not want a woman who will give notice after her first voyage, and whose sole idea is to see the world or gain a new experience. No work should be taken up in this spirit, and we hope that the pioneers of this movement will be women of good eductaion and steady character—women who will be an improvement in every way on the present stewardesses, or else where will be the advantage of their presence? And be it known that stewardesses are a very kindly race; they know that a pecuniary reward generally follows pleasant help, and they have learnt to be smiling and cheerful through all evils. But there is room for a very different class of workers amongst the sick. We all know what a grand thing training is, and even the best-intentioned stewardess could not make her patients a quarter so comfortable as an experienced nurse could. And it is not only sea-sickness that afflicts those who go on long voyages; other illnesses are often to the fore. Probably no nurse ever left a ship, though it was merely crossing to America, without having been called on to render professional aid to some one or other of the passengers. Only this morning a letter arrived at The Hospital Office from two nurses who had gone to Australia, and who throughout the whole voyage were engaged in nursing the many delicate passengers. The doctor parted with them with regret, saying that their services had been of the greatest aid to him.

If the companies tried the plan of carrying a trained nurse, and found it the great advantage we truly believe it would be, they might be persuaded in time to grant the nurse a slightly better position than that commonly held—make her head stewardess in fact, and grant her a few more comforts. But to the pioneers of this movement there will be many difficulties, we are sure, and though difficulties always dwindle if met in a cheerful, steadfast spirit, still we would not wish nurses to enter their names on the register without considering the *pros* and *cons*. The plan is one which should benefit all nurses and all travellers, and we confidently hope to have it working to the satisfaction of all concerned in a short time.

### Everybody's Opinion.

*[Correspondence on all subjects is invited. No communications can be entertained if the name and address of the correspondent is not given, or unless one side of the paper only be written on.]*

### A CORRECTION.

SISTER RUTH, of St. Barnabas Home Hospital, Eastbourne, writes: Much to my surprise I saw a letter in the *Standard* last week about Nurse Still, and as Nurse Still belongs to Sister Aimée's Home Hospital, I naturally take an interest in her, and cannot allow it to go forth that she belongs to St. John's Home. Neither myself nor Nurse Still had any intention of making public what was done simply and purely for the relief of the child, but since it has leaked out, I should like to say that she was presented with a beautiful gold cross, with a suitable inscription on it, in acknowledgment of her heroic conduct. The risk was especially great in her case, as she had only very recently recovered from a "hospital throat." I am pleased, however, to be able to state that she is quite well.

### REST AND EXERCISE.

A NURSE writes: Many thanks to "M. B." for confirming my statement on rest and exercise. May I be allowed to give a few painful facts that have taken place in a Private Nursing Institution? The place was instituted a few years ago by some nursing sisters. During this time it has been worked up patiently, and perfected for their own benefit, and a certain amount of consideration was shown to the nurses, who were working hard in order to obtain this perfection. But now the institution is known, and the nurses have been busily employed, the owners show a vast amount of independence. Until lately, the institution was generally known to the staff as the "Home"; but since a few rules have been pasted about the house to the effect "that no nurse is allowed to enter her room without permission," &c., it can be thought of as a home no longer. When the nurse goes in from her case, instead of getting a welcome, and, in case of a favourable report, a word of praise, she gathers from the sister's face that her food and pay, when not at work, are grudged her. Nearly all our nurses are of the same opinion as myself, and I have been persuaded by many to send these details, which perhaps appear trivial. Why do the private institutions charge an extra fee when their nurses are at mental or fever cases? They are very ready to reply, "Why, because the nurse gets overtaxed, and requires rest at the end of the case, and, indeed, she often gets ill; and this is why we have to charge an extra fee." True, the nurse does require a rest, but in nine cases out of ten she does not get it. If there is a case for her to go to she is sent, as a matter of course, rest or no rest. On the 7th of last November a nurse was suddenly called to her home in Inverness to her dying sister. She had been feeling ill herself for some time, but was nursing a mental case. Soon after she arrived home her sister was taken from them, and nurse herself fell ill. When she recovered her father fell ill, and thus they had quite a sick house. Nurse reported herself by letter, and sister wrote back to the effect that she was not to return until the doctor pronounced her fit for duty. Her father was still ill, but, with a keen sense of duty, she wrote that she would return January 12th. She left Inverness at nine p.m. on the 11th, and arrived at eleven a.m. on the 12th. Instead of finding a cup of tea and a welcome after all she had gone through, the sister at once set about giving her a scolding for not giving more notice of her intention to return, and informed her that her place had been filled up. "Very well," returned nurse, "that being the case, I can return at once." "Oh, you can go to bed," said sister, "and all I can do with you, after such a long holiday (!), is to send you to the first case that comes in." I wish some kind person would bring some plan forward to help nurses to combine to protect themselves from being imposed upon.

### NURSES AND STEWARDESSES.

S. A. writes: In the "Nursing Mirror" of the 26th ult. it is asked, "Would it serve a useful purpose to open a register for nurses who would like to go on voyages as stewardesses?" and that, "We have not heard of any nurse who is willing to be the pioneer of the movement." Now I believe that once it had become understood that the place of head stewardess on the large ocean-bound steamers were a suitable position for a thoroughly-trained nurse, as well as an educated woman, and one fitted to take the control of the passengers of all classes under her charge, and that she were offered a salary or percentage on the passengers, which would make it worth her while undertaking the hardships, sea-sickness, etc., I feel sure that it would not be difficult to find those willing to undertake it, more especially as it is so very difficult now to get good appointments, the competition is so great. I for one, though I have occupied the position of lady superintendent would now willingly, on the foregoing conditions, be a pioneer of the movement. I fully believe a nurse thoroughly trained in all branches to be as necessary as the doctor on a long voyage, where there may be cases of all kinds of illness under the greatest difficulties.

### A LACK OF COURTESY.

A MEMBER OF THE HOSPITALS ASSOCIATION writes: Being a nurse of some years standing, and happening to be staying in Hastings, I naturally much wished to see the splendid new hospital which has been recently built, and is called the "Hastings and East Sussex Hospital." Having ascertained that visitors were only admitted to see the hospital on Fridays between two and three p.m., I presented my-self at a quarter to three on Friday. I was informed by the porter that it was quite impossible that I should see the hospital, and in reply to my remark that the time allowed had not yet expired, he explained that no one was admitted who was not there precisely at two p.m., and that it was quite out of the question that I should see the hospital upon any other day. I venture to suggest that this is somewhat short-sighted policy on the part of the Committee of Management. Had I been a rich would-be subscriber instead of merely a nurse, I should certainly not feel that this hospital was commended to my liberality by this system of rendering inspection difficult, if not impossible, to anyone with only a limited space of time at their disposal.

### THE PENSION FUND.

F. F. writes: As one who feels grateful for the services rendered to him by a duly qualified nurse, I have read with great interest the excellent article by "Handclasp" in your issue of the 26th ult., as well as the comments upon it by Mr. Clifford. In considering the subject of these communications does not the question naturally arise, How far is it possible for nurses to set aside from their poorly paid earnings such an amount as will secure for them a decent provision for their later years? Can the remuneration received by the majority of them be regarded as a sufficient and fair return for the duties they have to perform—arduous and trying as they often are, especially in private nursing? If the remuneration is insufficient it ought in all fairness to be increased. The crowded ranks of nurses renders this impossible at present, I fear. Perhaps some of your corresponding nurses will offer some practical observations on this question.

### Appointments.

[It is requested that successful candidates will send a copy of their applications and testimonials, with date of election, to THE EDITOR, The Lodge, Porchester Square, W.]

THE HOSPITAL, AXMINSTER.—Miss R. R. Nichols has been appointed matron of this hospital. Miss Nichols was trained at the Leeds General Infirmary.

BOOTLE BOROUGH HOSPITAL.—Miss Catherine A. Macdonald, formerly of the Clinical Hospital, Manchester, has been appointed matron of the Bootle Borough Hospital.

### Lectures.

AT the Sanitary Institute, 74A, Margaret Street, on February 28th, Colonel W. Hope, on "The Metropolitan Sewage Question." Admission, 6d.

AT the Medical Societies' Rooms, 11, Chandos Street, on February 15th, at eight p.m., Mrs. Bedford Fenwick, on "The Matron." Admission by tickets from the Secretary of the B.N.A., 1s.

### Vacancies.

The following vacancies are announced:—

**Matrons.**

Children's Hospital, Mooredge, Newcastle; £50.

Northumberland County Asylum.

Milnthorpe Cottage Hospital; £20.

Jessop's Hospital for Women, Sheffield; £50.

**Superintendent.**

Perth Sick Poor Nursing Society.

**Nurses.**

Bedford Nurses' Institute; £24.

Llanelly Hospital; £20.

North London Consumption Hospital; £23.

Nurses' Home, Truro; £25.

Firs Home, Bournemouth (Night); £20.

Nursing Home, Rokeby Road, Brockley, S.E.; several.

Victoria Home, Bournemouth; several.

Swansea and South Wales Nursing Institute.

Essex and Colchester Hospital.

Huddersfield Infirmary; £24.

Tain Poorhouse; £25.

Southport Nurses' Home.

Bolton Infirmary.

Woolton Convalescent Home.

Home Hospital, Eastbourne.

Tewkesbury Hospital; £20.

Bridgworth Union; £25.

Blackheath Institute for Trained Nurses; £20.

Hospital for Epilepsy and Paralysis.

**District Nurses.**

Blackwater, Lyndhurst.

Leeds District Nurses' Home; £25. ward nurse

Glasgow Sick Poor Association.

Hospital for Epilepsy and Paralysis.

**Midwives.**

Glasgow Sick Poor Nursing Association.

**Probationers.**

Seamen's Hospital, Greenwich.

Coldstream Cottage Hospital.

Staffordshire Nurses' Institution, Stoke-on-Trent.

Lowestoft Hospital.

Brighton Boro' Fever Hospital.

Workhouse Infirmary Nursing Association.

Bath Nurses' Home; two.

### Notes and Queries.

**Queries.**

(106) *Florence Nightingale.*—Where shall I find the best life of Miss Nightingale?

**Answers.**

*Emily P.*—Apply at the nearest hospital and ask to see the matron; or apply to the Secretary, Workhouse Infirmary Nursing Association, 6, Adam Street, Strand, between eleven and noon.

*Mater.*—There is no fee. Age 25.

